# Effects of Lumbar Lordosis Angle on the Magnitude and Vertebral-Level Distribution of Anterior Cortical Bone Stress in the Lumbar Vertebral Bodies: A Finite Element Analysis

**DOI:** 10.3390/bioengineering13101116

**Published:** 2026-09-24

**Authors:** Keita Nishi, Daisuke Endo, Takashi Hasegawa, Shinichi Matsumoto, Masaru Itose, Madoka Makino, Kazunobu Saiki, Toshiyuki Tsurumoto, Keiko Ogami-Takamura

**Affiliations:** 1 Cadaver Surgical Training Center, School of Medicine, Nagasaki University, 1-12-4 Sakamoto, Nagasaki 852-8523, Japan; 2 Department of Macroscopic Anatomy, Graduate School of Biomedical Sciences, Nagasaki University, 1-12-4 Sakamoto, Nagasaki 852-8523, Japan; 3 Department of Health Sciences, Graduate School of Biomedical Sciences, Nagasaki University, 1-7-1 Sakamoto, Nagasaki 852-8520, Japan

**Keywords:** lumbar lordosis angle, anterior cortical bone of the vertebral bodies, finite element analysis

## Abstract

Lumbar lordosis influences load transmission in the lumbar spine, but its effects on stress in the anterior cortical bone of the vertebral bodies remain unclear. This study investigated how lumbar lordosis angle (LL) affects vertebral stress. A finite element model from T12 to the sacrum was constructed from computed tomography images of a cadaver from a 66-year-old male donor. Three models with identical bone geometry and material properties but LL values of 50°, 30°, and 15° were analyzed under a 300 N compressive load and the same load combined with a 5 N·m flexion moment. Mean von Mises stress and the 95th percentile of von Mises stress were evaluated in the anterior cortical bone of L1–L5, and vertebral-level distributions were normalized to L1. Under both loading conditions, the LL15° model showed higher von Mises stress than the LL50° and LL30° models at all vertebral levels. Adding the flexion moment further increased stress in all models. Normalized stress was lowest at L3 in the LL50° and LL30° models, whereas values in the LL15° model remained relatively close to L1 across L2–L5. These findings suggest that models with lower LL exhibit higher anterior cortical bone stress and altered vertebral-level stress distributions.

## 1. Introduction

Lumbar lordosis is a characteristic spinal morphology of the upright bipedal posture and plays an important role in sagittal spinal balance and load transmission [1,2]. Lumbar sagittal alignment affects the forces acting on spinal components, including intervertebral discs and facet joints, and is an important mechanical factor that determines load sharing in the lumbar spine [2,3].

Spinal sagittal alignment has been reported to change with age and spinal degeneration [4,5]. Intervertebral disc degeneration, loss of disc height, and spinal deformity may be accompanied by reduced lumbar lordosis and pelvic compensation, and vertebral deformity due to osteoporotic vertebral fractures has also been shown to affect spinal sagittal alignment [5,6]. Therefore, changes in lumbar lordosis and sagittal alignment may alter the mechanical loads acting on the lumbar spine.

The relationship between lumbar lordosis and spinal biomechanics has been investigated using finite element analyses and musculoskeletal models. Differences in lumbar lordosis angle (LL) have been reported to alter the compressive and shear forces acting on the intervertebral discs and the forces acting on the facet joints, thereby changing load sharing among spinal components [3,7,8]. In addition, finite element analyses using lumbar–pelvic models that reproduce different postures, including standing, chair sitting, and floor sitting, have shown that stresses in the intervertebral discs and vertebral cortical bone vary with changes in posture [9]. Such analyses are useful for comprehensively evaluating changes in lumbar–pelvic alignment associated with postural changes. However, because multiple alignment parameters, including lumbar lordosis, change simultaneously with posture, the relationship between differences in LL and the mechanical response of the vertebral cortical bone has not been sufficiently investigated.

Both cancellous and cortical bone play important roles in the mechanical strength of the vertebral body, and the vertebral cortical bone has been reported to contribute to load bearing [10]. In particular, the anterior region of the vertebral body is mechanically important in the development of anterior wedge deformity [11], and characteristic shear strains in the anterior cortical bone have also been reported after wedge compression fracture [12]. Therefore, evaluating stress in the anterior cortical bone is important for understanding the mechanical response of the vertebral body to changes in spinal alignment. However, the effects of differences in LL on the magnitude and vertebral-level distribution of stress in the anterior cortical bone of the vertebral bodies remain unclear.

Therefore, in the present study, finite element models with different LL values were constructed based on identical bone geometry and material properties, with reference to the range of LL values reported during standing, chair sitting, and floor sitting. This study aimed to determine the effects of differences in LL on stress in the anterior cortical bone of the lumbar vertebral bodies. In addition, vertebral-level stress distributions were compared under a compressive load alone and under a compressive load combined with a flexion moment. We hypothesized that a decrease in LL would increase stress in the anterior cortical bone of the vertebral bodies and alter its vertebral-level distribution.

## 2. Materials and Methods

### 2.1. Study Subject and CT Imaging

This study was approved by the Ethics Committee of the Nagasaki University Graduate School of Biomedical Sciences (approval no. 21092401-3; approved on 30 April 2026). Written informed consent for body donation and research use was obtained from the donor and his family prior to death.

A cadaver from a 66-year-old male donor (height, 165 cm) was used in this study. The cadaver was transported to Nagasaki University shortly after death, and computed tomography (CT) imaging from the head to the thighs was performed to obtain skeletal morphological data. CT images were obtained using a clinical multislice CT scanner (Activion 16; Toshiba, Tokyo, Japan). The scanning parameters were as follows: tube voltage, 120 kV; tube current, 300 mA; image matrix, 512 × 512 pixels; and slice thickness, 1.0 mm. The CT images were evaluated by a board-certified orthopedic surgeon, who confirmed the absence of spinal fractures and marked deformities.

### 2.2. Finite Element Model Construction

Finite element analysis was performed using MECHANICAL FINDER version 13.0 Extended Edition (Research Center of Computational Mechanics, Tokyo, Japan). The CT image data were imported into the software, and the built-in artificial intelligence segmentation function was used to extract the bone regions of each vertebra from T12 to L5. The sacral bone region was manually segmented. The extracted bone regions were converted into a three-dimensional skeletal model in the stereolithography (STL) format.

Based on the three-dimensional skeletal model, a finite element model comprising the cortical bone, cancellous bone, endplates, and intervertebral discs was constructed. The intervertebral discs were modeled separately as nucleus pulposus and annulus fibrosus. For the facet joints, the opposing articular surfaces were defined as frictionless contact elements with a coefficient of friction of 0 (Figure 1).

### 2.3. Material Properties and Mesh Settings

The material properties of each component of the finite element model were defined based on a previous study [13]. BMD derived from the CT data was not incorporated into the assignment of material properties; instead, fixed material properties were assigned to each component and applied consistently across all vertebral levels and LL models. The cortical bone and endplates were modeled using shell elements, whereas the cancellous bone, nucleus pulposus, and annulus fibrosus were modeled using solid elements. The annulus fibrosus was modeled as a homogeneous, isotropic, linear elastic material. The thicknesses of the cortical bone and endplates were set to 0.4 and 0.3 mm, respectively. The elastic modulus, Poisson’s ratio, and element type assigned to each component are listed in Table 1.

To assess the adequacy of the mesh size, a mesh convergence test was performed, consistent with previous lumbar spine finite element model development work [14]. Mesh sizes of 3.0, 2.0, and 1.5 mm were evaluated. The mean von Mises stress in the anterior cortical bone of the vertebral body, which was the primary outcome measure, was compared between the models. The percent change between the 2.0 and 1.5 mm models was 0.3%, which was within 5%. Based on this result, a mesh size of 2.0 mm was adopted in the present study. Element quality was additionally assessed using the maximum element aspect ratio.

### 2.4. Construction of Models with Different Lumbar Lordosis Angles

LL was defined, according to a previous study, as the angle between the extended lines of the superior surfaces of the L1 and S1 vertebral bodies in the sagittal plane [15]. Based on LL values reported during standing, chair sitting, and floor sitting [16,17], three finite element models were created with LL values of 50°, 30°, and 15°, designated as the LL50°, LL30°, and LL15° models, respectively (Figure 2). The mesh characteristics of the final LL50°, LL30°, and LL15° models are summarized in Table 2.

During mesh generation in MECHANICAL FINDER, the position and orientation of each vertebra were adjusted by translation and rotation while preserving the vertebral geometry until the target LL was achieved. The distribution of lordosis across the lumbar levels was adjusted with reference to the segmental lordosis reported by Bae et al. [17]. The segmental lordosis angles assigned to each intervertebral level are shown in Table 3.

The three-dimensional geometries of the annulus fibrosus and nucleus pulposus were created using the three-dimensional modeling software Metasequoia 4 (version 4.9.1, Tetraface Inc., Tokyo, Japan) and exported in STL format. The STL data were imported into MECHANICAL FINDER and manually positioned to fit each intervertebral space during the mesh generation for each model.

### 2.5. Loading and Boundary Conditions

For each model, two loading conditions were applied: a 300 N compressive load and a 300 N compressive load combined with a 5 N·m flexion moment. The loading magnitudes were selected with reference to previous lumbar finite element studies in which a 300 N axial load was used to represent simplified upper-body loading and a 5 N·m moment was applied to evaluate flexion–extension mechanics [18,19]. The combined loading condition was intended as a standardized mechanical scenario for evaluating the additional effect of flexion rather than as a direct reproduction of a specific physiological posture.

A 300 N compressive load was applied downward to the superior surface of T12 along the line connecting the centroid of the superior surface of T12 and the centroid of the inferior surface of L5. Under the flexion loading condition, a 5 N·m moment in the flexion direction was added to the 300 N compressive load.

Under all the analysis conditions, all degrees of freedom were constrained at the bilateral auricular surfaces of the sacrum (Figure 3). Two loading conditions were applied to each of the LL50°, LL30°, and LL15° models, resulting in a total of six finite element analyses.

### 2.6. Measurement Region and Outcome Measures

The anterior cortical bone of each vertebral body from L1 to L5 was designated as the measurement region. Using the lateral view in MECHANICAL FINDER, the anterior and posterior margins of the superior and inferior surfaces of the vertebral body were visually identified, and the midpoint of the anteroposterior diameter of each surface was determined. A dividing plane was manually positioned to pass through the midpoints of the superior and inferior surfaces, thereby dividing the vertebral body into anterior and posterior halves. The cortical bone located anterior to this plane was extracted as the measurement region (Figure 4). Cortical shell elements overlapping with the dividing plane were included in the anterior cortical bone measurement region. The pedicles and posterior elements were excluded from the measurement regions.

The mean von Mises stress and 95th percentile of von Mises stress were calculated for the measurement region of each vertebral body. In addition, the maximum value of the maximum principal stress and the minimum value of the minimum principal stress were determined and evaluated as indices of tensile and compressive stress, respectively.

Furthermore, to compare the vertebral-level stress distribution under the loading condition with the flexion moment, the mean von Mises stress and 95th percentile of von Mises stress at L2–L5 were divided by the corresponding L1 value for each LL model, yielding normalized values with L1 defined as 1.0. L1 was selected as a common cranial reference because it was the most cranial lumbar vertebral level included in the analysis, thereby allowing the relative caudal stress distribution from L1 to L5 to be compared consistently among the three LL models. The normalized values were used to characterize vertebral-level stress distribution, whereas absolute stress values were evaluated separately.

To assess the reproducibility of the manually defined measurement region, the same examiner redefined the measurement region for each vertebral body and recalculated the mean von Mises stress in the anterior cortical bone of each vertebral body. The percentage difference between the initial and repeated measurements ranged from 0.43% to 1.77% (mean 1.17%).

## 3. Results

### 3.1. Vertebral Stress Under a 300 N Compressive Load

The distribution of von Mises stress in each model under a 300 N compressive load is shown in Figure 5A. Compared with the LL50° and LL30° models, the LL15° model showed regions of relatively high von Mises stress in the anterior cortical bone of multiple vertebral bodies.

The mean von Mises stress and the 95th percentile of von Mises stress in the anterior cortical bone of the vertebral bodies are shown in Figure 6A,C, and detailed values for all stress measures are presented in Table 4. The mean von Mises stress ranged from 1.42–2.30 MPa in the LL50° model, 0.99–2.32 MPa in the LL30° model, and 2.07–2.81 MPa in the LL15° model. The LL15° model showed higher values than the LL50° and LL30° models at all vertebral levels from L1 to L5. In the LL50° and LL30° models, the maximum mean von Mises stress was observed at L5, whereas in the LL15° model, the maximum value of 2.81 MPa was observed at L2 and L5.

The 95th percentile of von Mises stress ranged from 2.70–4.14 MPa in the LL50° model, 2.01–3.69 MPa in the LL30° model, and 4.08–5.36 MPa in the LL15° model. Similar to the mean von Mises stress, the LL15° model showed higher values than the other two models at all vertebral levels from L1 to L5.

For the maximum value of the maximum principal stress, the LL15° model also showed higher values than the LL50° and LL30° models at all vertebral levels from L1 to L5. In contrast, the minimum value of the minimum principal stress was the lowest in the LL15° model at L2–L5, whereas at L1, it was the lowest in the LL50° model.

### 3.2. Vertebral Stress Under a 300 N Compressive Load Combined with a 5 N·m Flexion Moment

The distribution of von Mises stress in each model under a 300 N compressive load combined with a 5 N·m flexion moment is shown in Figure 5B. The distribution of von Mises stress differed among the LL50°, LL30°, and LL15° models, and the LL15° model showed relatively high-stress regions in the anterior cortical bone of each vertebral body from L1 to L5.

The mean von Mises stress and the 95th percentile of von Mises stress in the anterior cortical bone of the vertebral bodies are shown in Figure 6B,D, and detailed values for all stress measures are presented in Table 5. The mean von Mises stress ranged from 2.67–5.58 MPa in the LL50° model, 3.26–6.17 MPa in the LL30° model, and 5.22–6.91 MPa in the LL15° model. The LL15° model showed higher values than the LL50° and LL30° models at all vertebral levels from L1 to L5. In all three models, the maximum mean von Mises stress was observed at L5, with values of 5.58, 6.17, and 6.91 MPa in the LL50°, LL30°, and LL15° models, respectively.

The 95th percentile of von Mises stress ranged from 4.43–10.14 MPa in the LL50° model, 5.94–11.16 MPa in the LL30° model, and 11.09–13.65 MPa in the LL15° model. Similar to the mean von Mises stress, the LL15° model showed higher values than the other two models at all vertebral levels from L1 to L5.

For the maximum value of the maximum principal stress, the LL15° model also showed higher values than the LL50° and LL30° models at all vertebral levels from L1 to L5. The minimum value of the minimum principal stress was the lowest in the LL15° model at all vertebral levels from L1 to L5.

### 3.3. Vertebral-Level Distribution of Normalized Von Mises Stress Under the Flexion-Moment Loading Condition

To compare the vertebral-level stress distribution under a 300 N compressive load combined with a 5 N·m flexion moment, the mean von Mises stress and 95th percentile of von Mises stress were normalized to the corresponding L1 value, defined as 1.0, for each LL model (Figure 7).

For the mean von Mises stress, the normalized values decreased from L1 to L3 in both the LL50° and LL30° models, reaching minimum values of 0.54 and 0.61 at L3, respectively, and then increased from L4 to L5. In contrast, in the LL15° model, the normalized values at L2–L5 ranged from 0.83 to 1.10, and the reductions at L2–L4 were smaller than those in the LL50° and LL30° models (Figure 7A).

For the 95th percentile of von Mises stress, the LL50° and LL30° models also showed minimum values at L3, at 0.44 and 0.59, respectively, followed by an increase toward L5. In contrast, the normalized values at L2–L5 in the LL15° model ranged from 0.84 to 1.04, and as with the mean von Mises stress, the reductions at L2–L4 were smaller than those in the other two models (Figure 7B).

## 4. Discussion

### 4.1. Main Findings

This study investigated the effects of LL on stress in the anterior cortical bone of the vertebral bodies using finite element models with different LL values. Under the 300 N compressive load, the mean von Mises stress and 95th percentile of von Mises stress in the LL15° model were higher than those in the LL50° and LL30° models at all vertebral levels from L1 to L5. When a 5 N·m flexion moment was added, both stress measures increased at all vertebral levels in all LL models, and the LL15° model showed higher values than the other two models at all vertebral levels. Furthermore, the normalized von Mises stress was relatively low at L3 in the LL50° and LL30° models, whereas in the LL15° model, the values from L2 to L5 remained relatively close to that at L1. These findings suggest that differences in LL affect not only the magnitude of stress generated in the anterior cortical bone of the vertebral bodies but also the vertebral-level distribution.

### 4.2. Biomechanical Significance of Lumbar Lordosis

Lumbar lordosis is an important morphological feature associated with habitual upright bipedal locomotion in humans and contributes to positioning the trunk over the lower limbs [1,20]. It is also an important determinant of sagittal spinal alignment and contributes to mechanical stability during upright posture [2,21]. Previous finite element analyses and musculoskeletal modeling studies have shown that changes in lumbar lordosis alter the compressive and shear forces acting on the intervertebral discs, which are anterior spinal elements, as well as the forces acting on the facet joints, which are posterior spinal elements, thereby changing the load sharing between the anterior and posterior spinal elements [7,8,22]. In the present study, the LL15° model, which had the smallest lordosis, showed a higher von Mises stress in the anterior cortical bone of the vertebral bodies. This finding indicates that the LL15° configuration was associated with a greater mechanical burden on the anterior cortical bone of the vertebral bodies. However, because the present study primarily evaluated the anterior cortical bone of the vertebral bodies, facet joint forces and stresses in the posterior elements were not directly assessed. Therefore, changes in anterior–posterior load sharing cannot be determined from the present analysis alone. Future studies should evaluate the mechanical loads on both the anterior and posterior spinal elements to clarify how differences in lumbar lordosis are associated with anterior–posterior load sharing.

The addition of a 5 N·m flexion moment increased both the mean von Mises stress and the 95th percentile of von Mises stress at all vertebral levels in all three LL models. This indicates that superimposed flexion loading increases the mechanical burden on the anterior cortical bone even under the same compressive load. Importantly, the combined loading condition in the present study was not intended to reproduce a specific physiological posture. Rather, the 5 N·m flexion moment was applied as a standardized mechanical condition, based on loading magnitudes used in previous lumbar finite element studies, to examine how an additional flexion moment modifies the stress response across different LL configurations [18,19]. Therefore, the results should be interpreted as relative mechanical differences among the LL models under controlled loading conditions rather than as direct predictions of in vivo vertebral stress during a specific posture.

In addition, the vertebral-level distribution of stress in the anterior cortical bone of the vertebral bodies differed among the three LL models. Previous modeling studies have also demonstrated that sagittal geometry can influence segmental load distribution within the lumbar spine [7,8]. Under the loading condition with a flexion moment, the normalized von Mises stress was relatively low in the mid-lumbar spine, particularly at L3 in the LL50° and LL30° models, whereas the values at L2–L5 in the LL15° model remained relatively close to that at L1. This finding suggests that in the LL50° and LL30° models, the mechanical burden on the anterior cortical bone differs among vertebral levels, whereas these vertebral-level differences were smaller in the LL15° model. Furthermore, because the absolute stress under the flexion-moment loading condition was higher at all vertebral levels from L1 to L5 in the LL15° model than in the other two models, the LL15° configuration was associated with a greater mechanical burden on the anterior cortical bone across multiple vertebral levels, including the mid-lumbar spine.

The vertebral-level stress distribution observed in the present study differed in part from that reported by Cho et al. [9]. Under the flexion condition in their study, the mean von Mises stress in the L5 cortical bone was not the highest among L1–L5 in any of the four postures examined and was lowest at L5 during standing and floor sitting. In contrast, in the present study, the mean von Mises stress at L5 was the highest among L1–L5 in all LL models under the loading condition with a flexion moment. This difference may be attributable to differences in the measurement region and in the modeling and analysis conditions between the two studies. Cho et al. evaluated stress in the entire vertebral cortical bone, whereas the present study evaluated the cortical bone in the anterior half of the vertebral body. Therefore, direct comparison of the stress values and vertebral-level distributions between the two studies should be made with caution. In addition, Cho et al. used posture-specific models that included the pelvis, in which lumbar lordosis and sacral slope varied with posture and the pelvis–femur region was constrained, whereas in the present study, the sacrum was constrained and models with different LL values were constructed. These differences in lumbar–pelvic alignment and boundary conditions may have influenced load transmission across the lumbosacral junction. Differences in material assumptions and loading conditions should also be considered when comparing the two studies. The present model used homogeneous material properties assigned to each spinal component and applied a 300 N compressive load with a 5 N·m flexion moment, whereas Cho et al. applied a 300 N compressive load with a 10 N·m flexion moment and used different material definitions. Therefore, differences in absolute stress values between the two studies should not be attributed solely to differences in lumbar lordosis or posture. Furthermore, the facet joints were represented differently between the two studies. Cho et al. modeled the facet joints as material components with assigned elastic properties and used bonded contact between model components, whereas in the present study, the opposing articular surfaces were directly modeled as frictionless contact elements. In the present model, frictionless contact allows separation of the opposing articular surfaces and tangential sliding while transmitting compressive contact forces. These differences in facet-joint representation may alter the mechanical contribution of the facet joints to posterior load sharing and, consequently, the stress transmitted to the vertebral bodies. However, because facet-joint forces and posterior-element stresses were not directly evaluated and no sensitivity analysis comparing different facet-joint representations was performed, the extent to which this modeling difference contributed to the observed stress distribution cannot be determined from the present study.

### 4.3. Comparison with Reported Vertebral Bone Mineral Density Distribution

Bone tissue is known to adapt its mass and structure to the mechanical environment through bone modeling and remodeling [23,24], and experimental studies have shown that bone mass changes according to the magnitude of the mechanical strain [25]. Bone mineral density (BMD) also differs among skeletal sites within the same individual, and local factors, such as the loading environment, bone microstructure, and mode of ossification, may contribute to these site-specific differences [26].

In the lumbar spine, BMD exhibits a vertebral-level-dependent distribution. In a study of 136 Japanese participants in which the trabecular BMD of the vertebral bodies was measured using quantitative computed tomography (QCT), no statistically significant differences in BMD were found from T12 to L5, although a vertebral-level-dependent distributional trend was observed [27]. Another QCT study reported that BMD decreased from L1 to L3 and then increased from L4 to S1 [28]. In the LL50° model of the present study, the normalized von Mises stress under the loading condition with the flexion moment was also relatively low in the mid-lumbar spine, particularly at L3, and increased again toward L5. However, BMD was not incorporated as a vertebral-level-specific material property in the present model; therefore, the calculated stress distribution reflects the modeled geometry, alignment, and loading conditions under identical material assumptions rather than site-specific differences in bone quality. Site-specific variations in BMD and bone quality could alter local stiffness and thereby influence vertebral stress distribution. Accordingly, the qualitative similarity between the stress distribution observed in the present study and previously reported BMD distributions should be regarded as hypothesis-generating rather than evidence of a direct relationship. Moreover, the present study evaluated stress in the anterior cortical bone, whereas the cited QCT studies primarily evaluated trabecular BMD. Further studies incorporating vertebral-level-specific bone properties are required to clarify the relationship between mechanical stress distribution and BMD distribution.

### 4.4. Potential Relevance of Reduced Lumbar Lordosis to Vertebral Fracture Mechanics

In clinical practice, BMD is widely used as an important indicator for assessing osteoporosis and fracture risk; however, spinal sagittal alignment has also been reported to be associated with vertebral fracture risk [29,30]. The changes in the magnitude and vertebral-level distribution of stress in the anterior cortical bone of the vertebral bodies associated with differences in LL observed in the present study suggest that differences in spinal sagittal alignment may modify the mechanical environment of the vertebral bodies. Such mechanical effects may be relevant to understanding biomechanical factors associated with vertebral fracture, although fracture risk itself was not evaluated in the present study.

Osteoporotic vertebral fractures can result in morphological changes, such as vertebral wedging and local kyphosis, thereby altering spinal sagittal alignment [6,31]. Furthermore, the prevention of subsequent vertebral fractures after an initial fracture is an important clinical issue [32,33]. Based on the present findings, postfracture changes in spinal alignment could alter the mechanical environment of the remaining vertebral bodies. This may represent a potential biomechanical mechanism linking postfracture alignment changes to subsequent vertebral loading; however, the present study does not demonstrate a causal relationship with subsequent vertebral fracture occurrence.

Future studies incorporating individual differences in BMD, bone geometry, and spinal alignment, together with models that reproduce postfracture morphological changes and include bone failure criteria, are needed to determine whether these mechanical changes are associated with subsequent vertebral fracture. Longitudinal clinical studies will also be required to establish whether changes in sagittal alignment are associated with subsequent fracture occurrence.

### 4.5. Limitations

This study had several limitations. First, a finite element model constructed from a single cadaver was used, and interindividual differences in bone geometry and BMD were not considered in the analysis. In addition, vertebral-level variations in BMD were not incorporated into the material properties of the model.

Second, LL varied while maintaining identical bone geometry and material properties; therefore, the models did not reproduce the morphological changes that occur with actual aging or spinal degeneration. Although this controlled parametric approach allowed comparison of different LL configurations while minimizing confounding effects from differences in bone geometry and material properties, the findings may not fully represent naturally occurring variations in spinal alignment. In addition, because the segmental distribution of lordosis differed among the three LL models, the present study cannot fully distinguish the effect of total LL from the contribution of specific segmental lordosis patterns.

Third, the loading conditions were limited to a 300 N compressive load and a 5 N·m flexion moment, which were used as standardized mechanical conditions rather than to reproduce the full range of physiological loading conditions. Therefore, the present findings cannot be directly generalized to other loading magnitudes or movement directions. To maintain a simplified parametric framework for comparing the LL configurations under standardized loading conditions, ligamentous structures, muscle–tendon forces, and intra-abdominal pressure, which contribute to physiological spinal stability and load sharing, were not incorporated into the model; their omission may have affected both the absolute stress magnitudes and, potentially, the relative differences among the LL models because the contributions of these structures can vary with spinal alignment and loading condition [34,35]. Future studies incorporating these structures and more physiologically representative loading conditions are needed to determine whether the stress patterns observed in the present study are maintained under more realistic biomechanical conditions.

Fourth, the annulus fibrosus was modeled as a homogeneous, isotropic, linear elastic material. This simplification does not reproduce the anisotropic and nonlinear mechanical behavior associated with the fibrous architecture of the annulus fibrosus [36,37] and may have influenced load transfer through the intervertebral discs. Future studies incorporating anisotropic, fiber-reinforced, or hyperelastic constitutive models are needed to evaluate the influence of more physiologically realistic disc behavior on vertebral stress distribution.

Fifth, stress in the anterior cortical bone of the vertebral bodies was the primary focus, and bone failure or fracture was not evaluated. Because no bone strength or failure criteria were incorporated into the model, the calculated stress values should not be interpreted as direct indicators of fracture occurrence or fracture risk. Future studies should include multiple subjects to account for interindividual variability and incorporate subject- and vertebral-level-specific bone properties and bone failure criteria into the analyses.

## 5. Conclusions

The present study showed that models with lower LL exhibited increased stress in the anterior cortical bone of the vertebral bodies and altered the vertebral-level distribution. The addition of a flexion moment further increased the stresses. These findings suggest that lumbar lordosis may be an important factor influencing the mechanical environment of the vertebral bodies and may provide a biomechanical basis for future studies investigating how spinal sagittal alignment influences vertebral loading. Further studies incorporating bone quality and failure criteria are required to determine whether these mechanical effects are related to vertebral fracture occurrence.

## Figures and Tables

**Figure 1:**
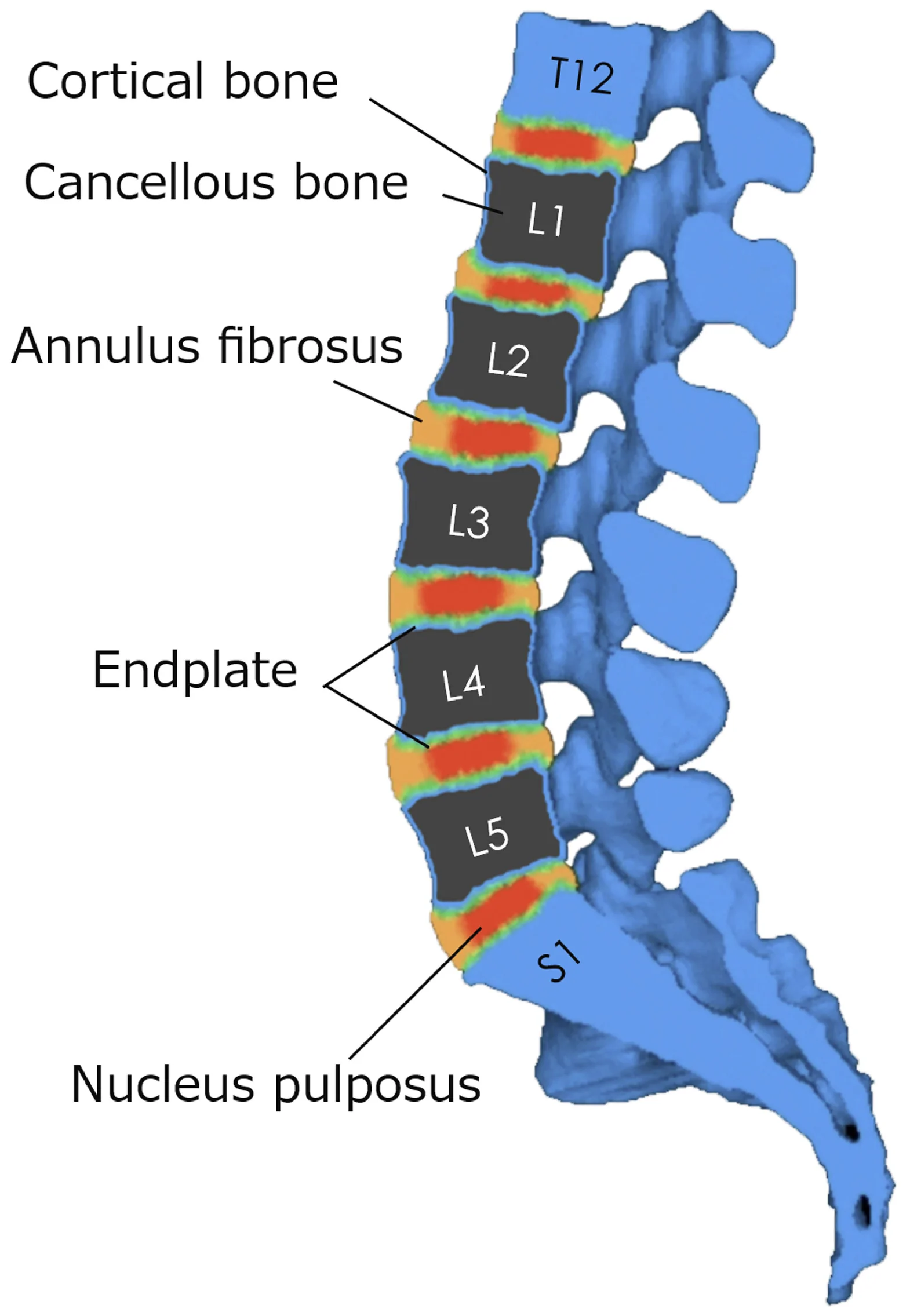
Main components of the finite element model. The model consisted of the cortical bone, cancellous bone, endplates, annulus fibrosus, and nucleus pulposus. The colors of the anterior spinal elements from L1 to L5 indicate the following components: blue, cortical bone; dark gray, cancellous bone; green, endplates; orange, annulus fibrosus; and red (dark orange), nucleus pulposus.

**Figure 2:**
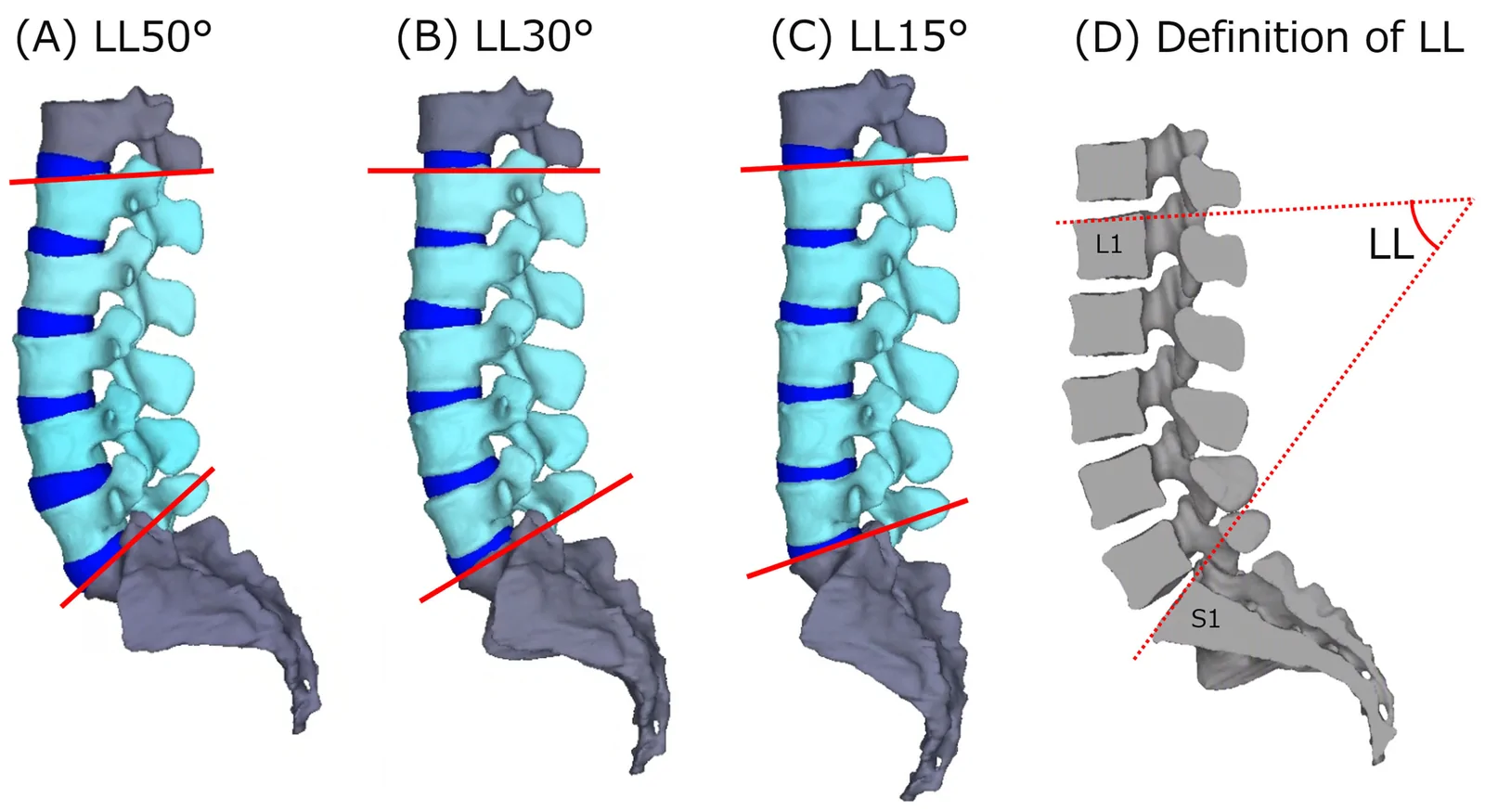
Finite element models with different lumbar lordosis angles and definition of the lumbar lordosis angle: (**A**) LL50° model; (**B**) LL30° model; and (**C**) LL15° model. (**D**) Lumbar lordosis angle (LL) was defined as the angle between the red lines extending along the superior surfaces of the L1 and S1 vertebral bodies in the sagittal plane.

**Figure 3:**
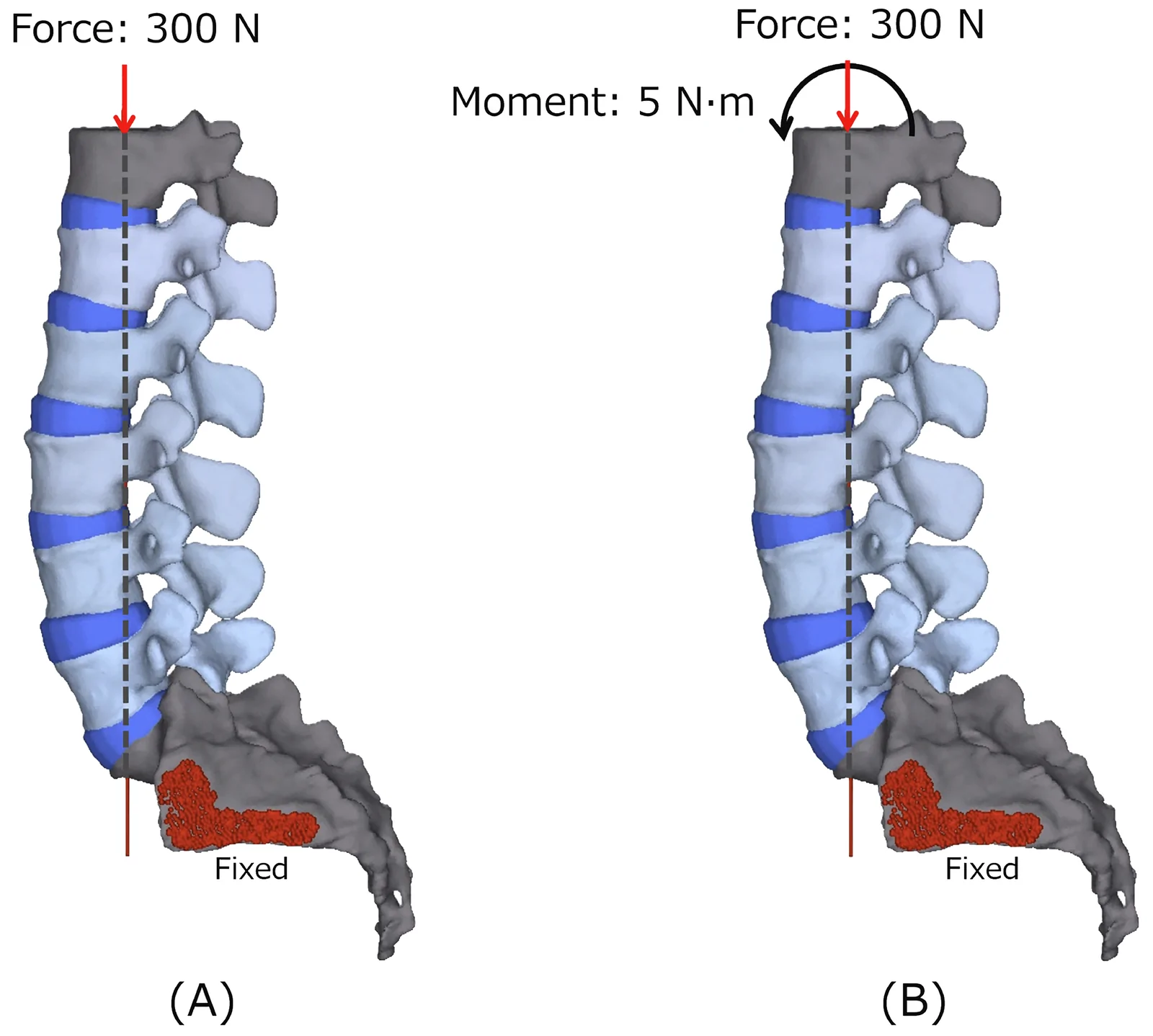
Loading and boundary conditions in the finite element analysis: (**A**) 300 N compressive load. (**B**) 300 N compressive load combined with a 5 N·m flexion moment. The red downward arrows indicate the 300 N compressive load, and the curved black arrow indicates the 5 N·m flexion moment. The gray dashed line indicates the direction of the compressive load, connecting the centroid of the superior surface of T12 and the centroid of the inferior surface of L5. The red regions on the sacrum indicate the constrained surfaces, where all degrees of freedom were fixed.

**Figure 4:**
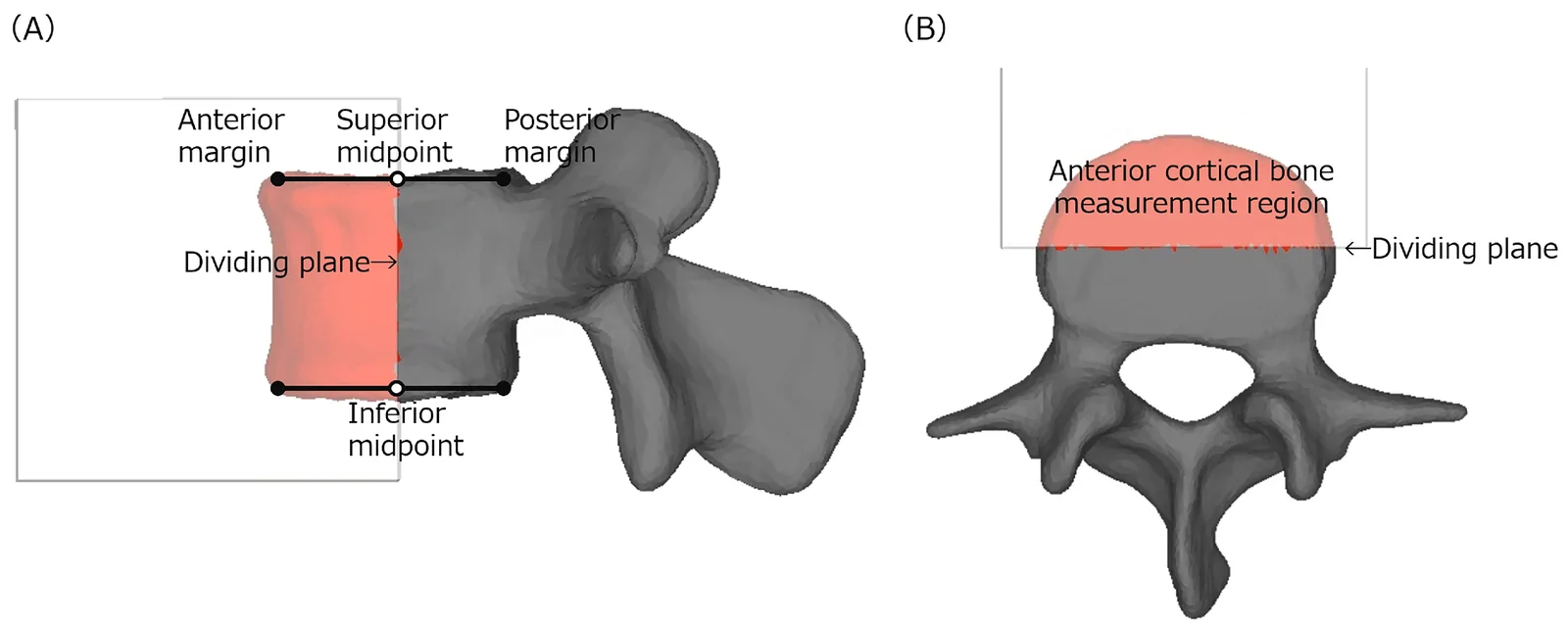
Definition of the measurement region in the anterior cortical bone of the vertebral body. (**A**) In the lateral view of MECHANICAL FINDER, the anterior and posterior margins of the superior and inferior surfaces of the vertebral body were identified, and the midpoint of the anteroposterior diameter of each surface was determined. A dividing plane was manually positioned to pass through the midpoints of the superior and inferior surfaces, thereby dividing the vertebral body into anterior and posterior halves. (**B**) The cortical bone located anterior to the dividing plane was extracted as the anterior cortical bone measurement region.

**Figure 5:**
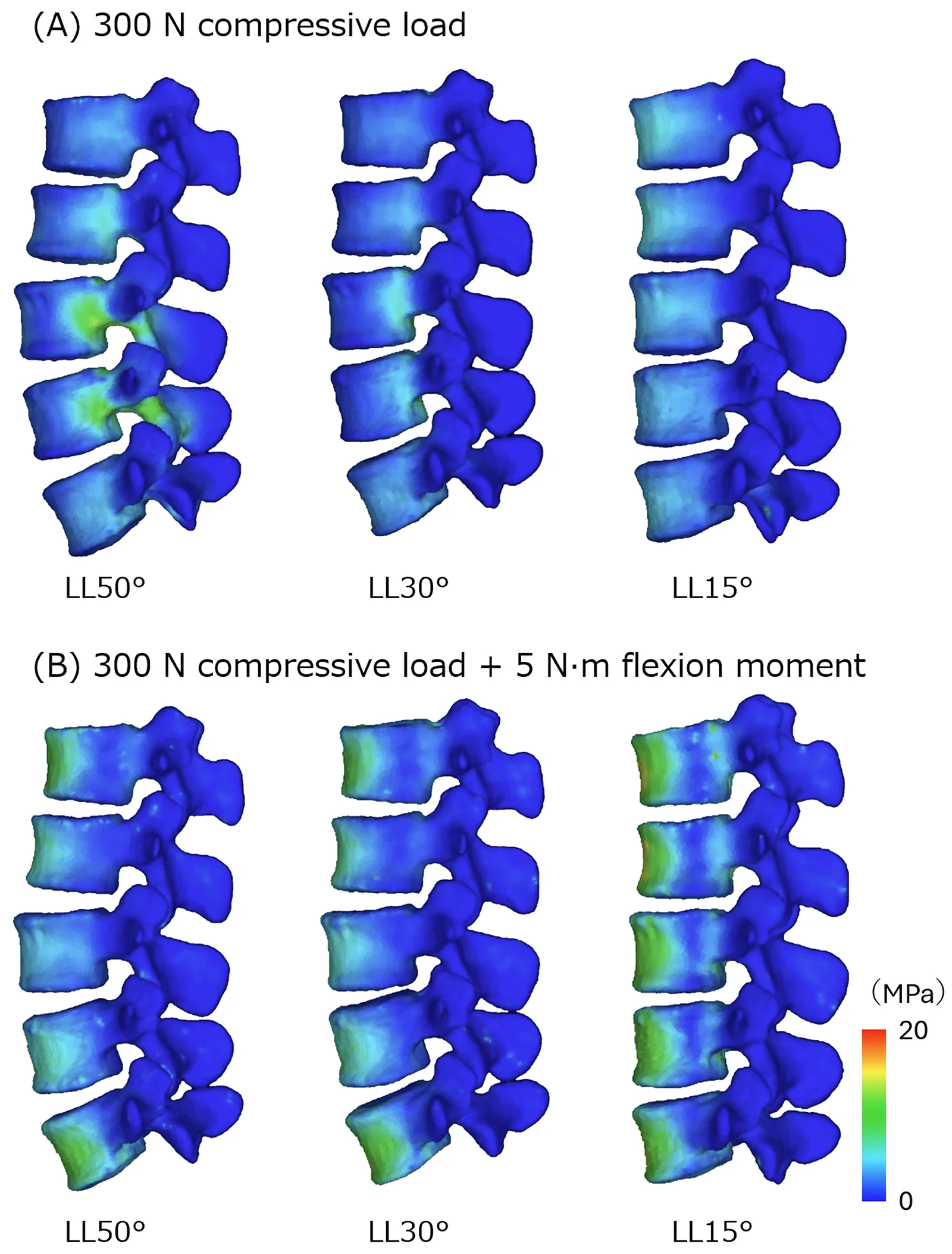
Distribution of von Mises stress for each loading condition. (**A**) 300 N compressive load. (**B**) 300 N compressive load combined with a 5 N·m flexion moment. The same stress scale (0–20 MPa) was used for all the models.

**Figure 6:**
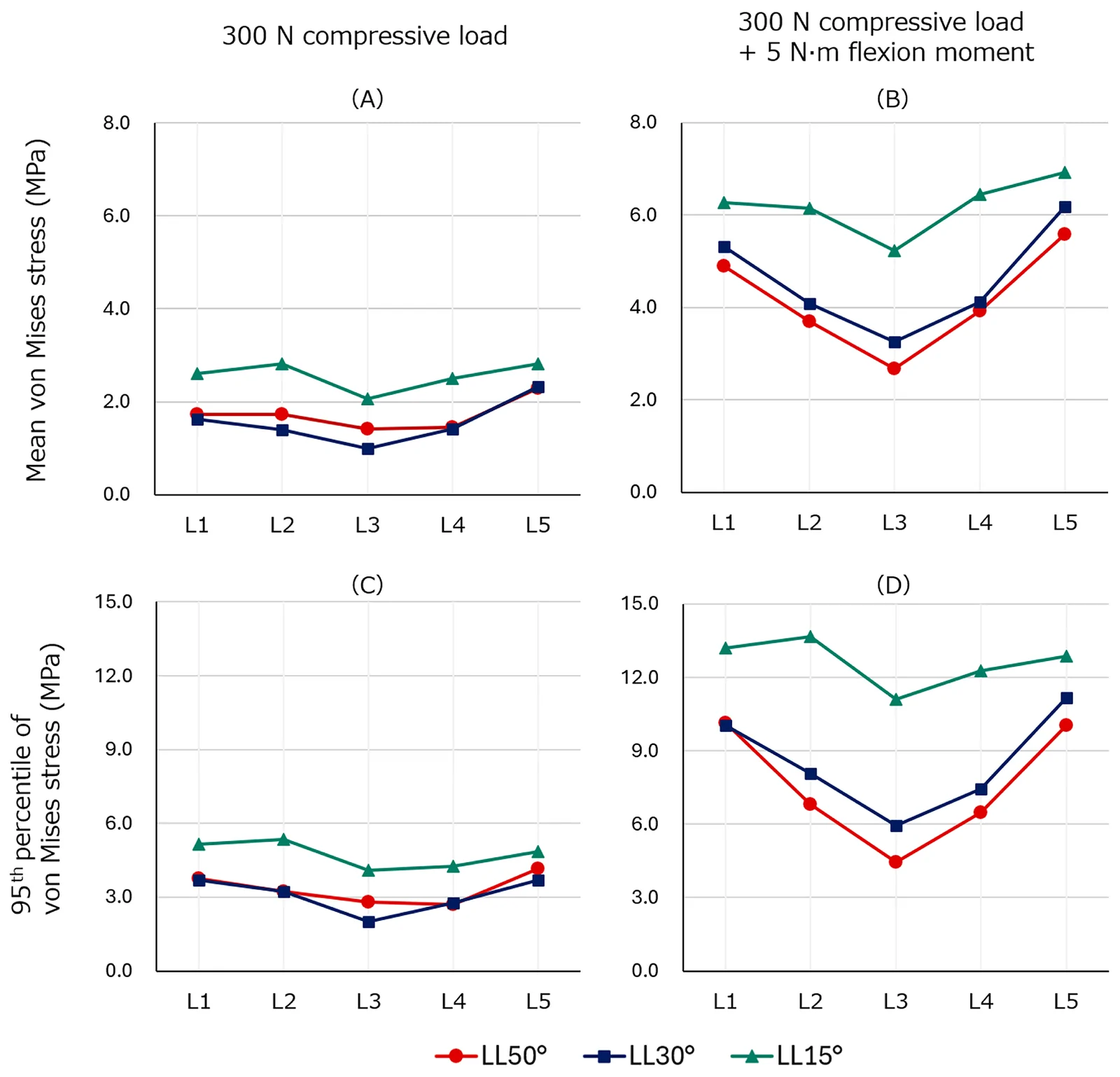
Von Mises stress in the anterior cortical bone of the lumbar vertebral bodies under different loading conditions. (**A**) Mean von Mises stress under a 300 N compressive load. (**B**) Mean von Mises stress under a 300 N compressive load combined with a 5 N·m flexion moment. (**C**) 95th percentile of von Mises stress under a 300 N compressive load. (**D**) 95th percentile of von Mises stress under a 300 N compressive load combined with a 5 N·m flexion moment.

**Figure 7:**
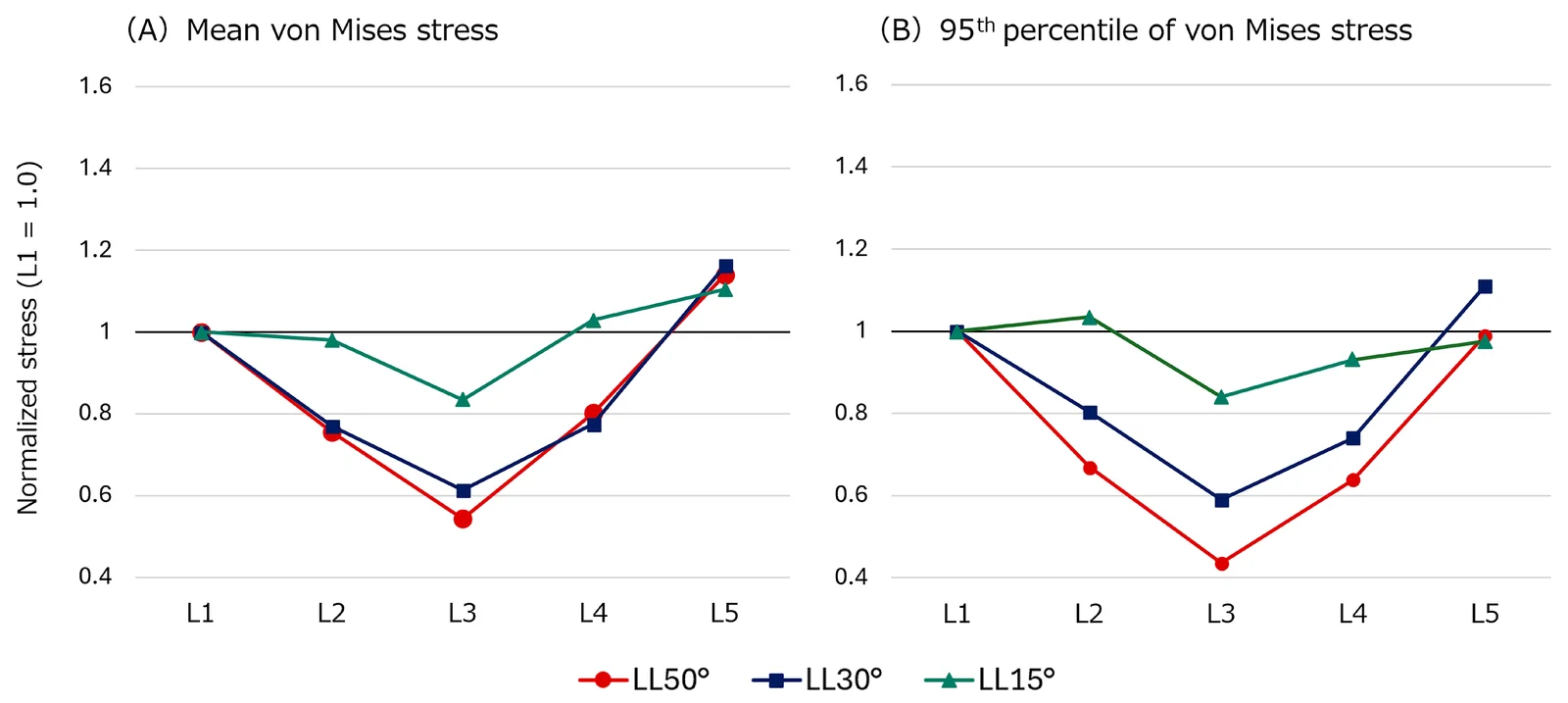
Vertebral-level distribution of normalized von Mises stress under a 300 N compressive load combined with a 5 N·m flexion moment. (**A**) Mean von Mises stress. (**B**) 95th percentile of von Mises stress. For each LL model, the stress values at L2–L5 were normalized to the corresponding value at L1, with L1 defined as 1.0.

**Table 1:** Material properties and element settings for the components of the finite element model.

Component	Elastic Modulus (MPa)	Poisson’s Ratio	Element Type
Cortical bone	12,000	0.3	Shell (thickness = 0.4 mm)
Cancellous bone	200	0.25	Solid
Endplate	1000	0.3	Shell (thickness = 0.3 mm)
Nucleus pulposus	1	0.49	Solid
Annulus fibrosus	4.2	0.45	Solid
Facet joint	—	—	Contact elements

**Table 2:** Mesh characteristics of the final finite element models.

Model	Number of Nodes	Number of Elements	Maximum Element Aspect Ratio
LL50°	225,802	1,278,963	9.13
LL30°	209,405	1,180,245	9.16
LL15°	219,869	1,243,385	9.16

The total number of elements includes solid, shell, and contact elements.

**Table 3:** Segmental lordosis angles in the LL50°, LL30°, and LL15° models.

Level	LL50°	LL30°	LL15°
L1–L2	5.5°	6.0°	5.0°
L2–L3	4.5°	3.0°	0.7°
L3–L4	7.0°	5.0°	1.0°
L4–L5	13.0°	6.0°	0.8°
L5–S1	20.0°	10.0°	7.5°
Total LL	50°	30°	15°

**Table 4:** Stress measures in the anterior cortical bone of the lumbar vertebral bodies under a 300 N compressive load.

Model	Vertebral Level	Mean von Mises Stress	95th Percentile of von Mises Stress	Maximum Value of Maximum Principal Stress	Minimum Value of Minimum Principal Stress
LL50°	L1	1.74	3.76	5.89	−5.83
	L2	1.73	3.24	5.08	−4.33
	L3	1.42	2.80	4.30	−3.04
	L4	1.44	2.70	3.84	−3.76
	L5	2.30	4.14	4.48	−4.96
LL30°	L1	1.62	3.69	4.89	−5.75
	L2	1.40	3.21	4.70	−5.41
	L3	0.99	2.01	3.22	−3.11
	L4	1.42	2.77	4.20	−4.85
	L5	2.32	3.68	3.54	−4.83
LL15°	L1	2.61	5.13	6.91	−5.64
	L2	2.81	5.36	6.46	−6.45
	L3	2.07	4.08	5.30	−4.50
	L4	2.50	4.27	4.63	−5.38
	L5	2.81	4.84	5.08	−6.06

All stress values are expressed in MPa.

**Table 5:** Stress measures in the anterior cortical bone of the lumbar vertebral bodies under a 300 N compressive load combined with a 5 N·m flexion moment.

Model	Vertebral Level	Mean von Mises Stress	95th Percentile of von Mises Stress	Maximum Value of Maximum Principal Stress	Minimum Value of Minimum Principal Stress
LL50°	L1	4.90	10.14	12.66	−15.45
	L2	3.70	6.80	9.32	−8.86
	L3	2.67	4.43	10.89	−7.68
	L4	3.93	6.47	6.83	−9.55
	L5	5.58	10.05	10.91	−15.03
LL30°	L1	5.31	10.05	13.62	−11.84
	L2	4.09	8.08	11.45	−13.64
	L3	3.26	5.94	7.71	−6.31
	L4	4.12	7.44	7.91	−13.54
	L5	6.17	11.16	13.11	−15.78
LL15°	L1	6.26	13.19	18.67	−15.98
	L2	6.13	13.65	18.36	−18.89
	L3	5.22	11.09	14.48	−13.14
	L4	6.44	12.27	14.17	−17.89
	L5	6.91	12.87	15.00	−18.71

All stress values are expressed in MPa.

## Data Availability

The data supporting the findings of this study are available from the corresponding author upon reasonable request, subject to ethical restrictions.

## Abbreviations

The following abbreviations are used in this manuscript:

LL	Lumbar lordosis angle
CT	Computed tomography
STL	Stereolithography
BMD	Bone mineral density
QCT	Quantitative computed tomography
